# The importance of being persistent: The first true resident gut symbiont in Drosophila

**DOI:** 10.1371/journal.pbio.2006945

**Published:** 2018-08-02

**Authors:** Dali Ma, François Leulier

**Affiliations:** Institut de Génomique Fonctionnelle de Lyon, Université de Lyon, Ecole Normale Supérieure de Lyon, Centre National de la Recherche Scientifique, Université Claude Bernard Lyon 1, Unité Mixte de Recherche 5242, Lyon, France

## Abstract

In the animal kingdom, nutritional mutualism is a perpetual and intimate dialogue carried out between the host and its associated gut community members. This dialogue affects many aspects of the host’s development and physiology. Some constituents of the animal gut microbiota can stably reside within the host for years, and such long-term persistence might be a prerequisite for these microbes to assert their beneficial impact. How long-term persistence is established and maintained is an interesting question, and several classic model organisms associated with cultivable resident strains are used to address this question. However, in *Drosophila*, this model has long eluded fly geneticists. In this issue of *PLOS Biology*, Pais and colleagues present the most rigorous and comprehensive demonstration to date that persistence and gut residency do take place in the digestive tract of *Drosophila melanogaster*. This natural gut isolate of *Acetobacter thailandicus* stably colonizes the adult fly foregut, accelerates larval maturation, and boosts host fecundity and fertility as efficiently as the known laboratory strains. The discovery of such stable association will be a boon for the *Drosophila* community interested in host–microbiota interaction, as it not only provides a novel model to unravel the molecular underpinnings of persistence but also opens a new arena for using *Drosophila* to study the implications of gut persistence in evolution and ecology.

The astounding biodiversity on Earth is a testament to the evolutionary triumph of symbiosis. However, it was not until recently that the revolution in sequencing technologies has fortuitously transformed the studies of symbiosis from a somewhat esoteric endeavor to a unifying theme of biology [1–3]. One of the most ancient and prevalent forms of symbiosis is nutritional mutualism, forged by the animal host and its gut bacterial symbionts. In this context, the host furnishes a nutrient-rich and predation-free habitat for the symbionts to thrive, and in return, with their vast and versatile metabolic toolkit, the symbionts help the host extract and metabolize nutrients from food that the host cannot otherwise utilize. Nutritional mutualism is pervasive in nature, and it profoundly impacts host biology, from development and physiology to behavior and ecological adaptation (reviewed in [4], [5], [6], and [7]).

Nutritional mutualism requires constant and intimate association between the host and its bacteria symbiont. It takes on several modalities. An extreme form of such modalities manifests as obligate endosymbionts persisting within the host cell as a dedicated organ, such as the bacteriocytes found in the pea aphids [8], tsetse flies [9], and weevils [10]. This highly specialized mutualistic association is vertically transmitted and maintained from generation to generation. In vertebrates that do not harbor bacteriocyte-like structures, constant association is made possible by colonization and long-term persistence. For example, *Bacteroides fragilis*, an abundant species found in the large intestines in most mammals, aggregates into clusters buried deep within the mucus layer of the intestine, and their adherence is reinforced by the interaction between host immunoglobulin A (IgA) and bacterial surface polysaccharides [11]. This intricate mechanism may account for how a commensal strain can persist within the host for years [12]. Furthermore, in some animals such as insects, many gut community members are transient, and sometimes a resident microbiota is lacking [13, 14]; yet, if the microbes are ubiquitously present in the food that the host consistently ingests, sometimes through coprophagy, constant association can be sustained. Nutritional mutualism in *Drosophila melanogaster*, a classic genetic model organism, is a perfect representation of such transient and dynamic association. A fly host ingests large quantities of bacteria via food intake and excretes them through either defecation or regurgitation; the purged bacteria then quickly re-inoculate and multiply on the food substrate where the fly refeeds [15, 16]. This “farming” cycle efficiently perpetuates mutualistic interactions between the fly larvae and one of its prevalent model gut commensals, *Lactobacillus plantarum* [16].

From a decade of independent studies by several research groups, the accumulated evidence that excludes the presence of a long-term, resident gut commensal in *D*. *melanogaster* prevailed. First, in the standard laboratory environment, the gut community of *Drosophila* is acquired through food. Within the first hour of eclosion, only 10% of the teneral flies carry barely detectable amounts of gut bacteria. The gut colony-forming units (CFUs) increase steadily after 24 hours, but every transfer to fresh, sterile food imposes a drastic population bottleneck on the gut bacteria carried over by the host, and frequent transfers can virtually render the environment germ free [15]. Proliferation therefore takes place in the food rather than in the gut. The acquired microbes are low in diversity. They fall into two major bacteria phylotypes, dominated by the *Acetobacter* and *Lactobacilli* species [17, 18]. Depending on the diet, developmental stage, age, and health status, the abundance and composition of the gut communities change and evolve and vary unpredictably from individual to individual [18–21]. Compared to the laboratory strains, the natural fly gut communities are much more complex. Several deep 16S RNA-sequencing experiments were conducted on natural gut bacteria communities isolated from flies of different Drosophilids caught in different geographical locations and fed on different food sources. The end conclusion is that in both the lab-reared and wild-captured flies, no single bacteria strain is present in all flies at any given time and no common strains are found between laboratory and wild flies [20, 22–24]. Altogether, the lack of persistence of the gut population in laboratory flies and the absence of a “core microbiota” strain strongly suggest that there is no stable symbiont in the *Drosophila* digestive tract.

However, lacking a resident gut commensal strain has not held the fruit fly back from contributing to host–microbiota research. Thanks to the sophisticated fly genetic toolkit and its easily cultivable gut bacteria species, studies employing germ-free and gnotobiotic flies have generated detailed phenomenological and molecular descriptions of nutritional mutualism with unstable association in the context of juvenile growth, undernutrition [16, 25, 26], metabolic rewiring [27, 28], transcriptional regulation[19, 29, 30], and dysbiosis and aging [31–33]. Furthermore, novel and unexpected findings can turn up when working with transient gut community members. In a long-term experimental evolution study to understand how the gut microbiota promotes larval growth during chronic undernutrition, a *Lactobacillus* strain transformed itself from a poor growth promoter to an efficient one in just one fly generation simply by adapting to the food [34]. This observation brings important insight to understanding the initial establishment of facultative nutritional mutualism but also illustrates that even if its gut microbiota is transient, volatile, and low in diversity, *Drosophila* is still a prolific model to study the molecular mechanism and evolutionary origin of nutritional symbiosis.

However, the analyses from natural *Drosophila* isolates strongly insinuate that persistence may still be “out there,” that one has not looked hard enough. For example, *D*. *nigrospiracula* feeding on the giant cactus in the Sonoran Desert carry a gut microbiota that is extremely different from what they feed on [35]. Moreover, a wild isolate of *L*. *plantarum* strain can easily reach and maintain constant high titers in the fly gut [36]. Now, in this issue of *PLOS Biology*, an elegant study by Pais and colleagues provides compelling evidence that stable association between *Drosophila* and some of its gut microbial partners does exist in nature [37]. They isolated gut bacteria from wild *D*. *melanogaster* trapped with figs and assessed their diversity and ability to persist in the fly gut. Specifically, with a stringent “stability protocol,” they systematically subjected single flies associated with either lab- or wild-derived gut communities to a frequent transfer schedule that minimized environmental contamination and extracted bacteria from individual fly guts for CFU counts and 16S RNA sequencing on the isolated colonies. First, the wild-fly–derived gut bacteria were much more diverse than that found in the standard laboratory stain, *w*^*1118*^: 35 operation taxonomic units (OTUs) versus 2. At the end of the protocol, the wild communities maintained relatively constant CFU counts, whereas the CFUs of the standard laboratory strains decreased by 1,000-fold. These initial observations confirm several previous findings. What is unexpected is that among the 35 wild bacterial OTUs, the authors identified a natural isolate of *Acetobacter thailandicus* that, when introduced to axenic flies, can stably and persistently colonize the *Drosophila* digestive tract.

To unequivocally prove stable and persistent colonization is no trivial feat. The aforementioned farming mechanism can maintain high and constant gut bacteria level by rapid transit and proliferation on the food, not necessarily persistence in the intestinal tract. In fact, when subjected to the stability protocol, *A*. *thailandicus* can effectively repopulate the food substrate, whereas the laboratory strain used in this study, *Acetobacter* OTU 2753, cannot. To firmly pin down that the CFU counts originate from the fly gut, not the food, the author slightly altered the stability protocol by enclosing a single fly in a cage with six food caps, thereby increasing the available food surface area 100 times. Consequently, the chance that the fly reingests the same bacteria that it has excreted is close to nil. At the end of the experiment, the control fly gut titers drop 10,000-fold and 50% of the control hosts yield no detectable CFUs at all, but the *A*. *thailandicus* level remains high and constant in almost 100% of the associated hosts. In an ingenious growth assay, five axenic fly “chasers” were introduced to the same cage where the *A*. *thailandicus* monoassociated host forages, and none of the chasers had acquired any detectable bacteria in the gut after 24 hours of cohousing, indicating that high gut titer of *A*. *thailandicus* is not a result of reinfection through food but of gut persistence.

Where, then, does *A*. *thailandicus* reside in the fly gut? The adult fly digestive tract is a highly compartmentalized structure comprising the foregut, the midgut, and the hindgut (Fig 1). In insects such as arboreal termites and cockroaches, both the foregut crop and the hindgut provide major habitats for the resident microorganisms [38]. In *Drosophila*, the diverticulated crop is a unique adult structure that develops de novo during metamorphosis from the imaginal rings in the larval proventriculus. It is a food storage organ innervated by the insulinergic neurons, a muscular pump whose volume and activities are controlled by various factors such as dietary sugar content, hemolymph osmolarity and concentration, and locomotion [39, 40]. In a previous study, Obadia and colleagues also reported that a wild *L*. *plantarum* isolate attaches to the cardia, the foregut portion of the proventriculus [36]. On the first day of the stability protocol, the titers of *A*. *thailandicus* in monoassociated flies are evenly distributed along the entire digestive tract. Five days later, the bacteria populations in the midgut and hindgut regions vanish, but stable CFUs are consistently yielded by what remain in the crop and proventriculus, two separate structures forming the foregut. Through detailed fluorescent in situ hybridization (FISH) analysis, transmission electron microscopy (TEM) imaging, and stainings that mark live and dead bacteria, the authors exhaustively demonstrated that *A*. *thailandicus* predominantly localizes to the crop and proventriculus, where it probably binds to chitin filaments, forms clusters through fimbrae, and likely undergoes cell division. To date, these are the most comprehensive and rigorous demonstration that adult *Drosophila* can carry a natural resident gut bacterial population that stably and persistently colonize its digestive tract (Fig 1).

**Fig 1:**
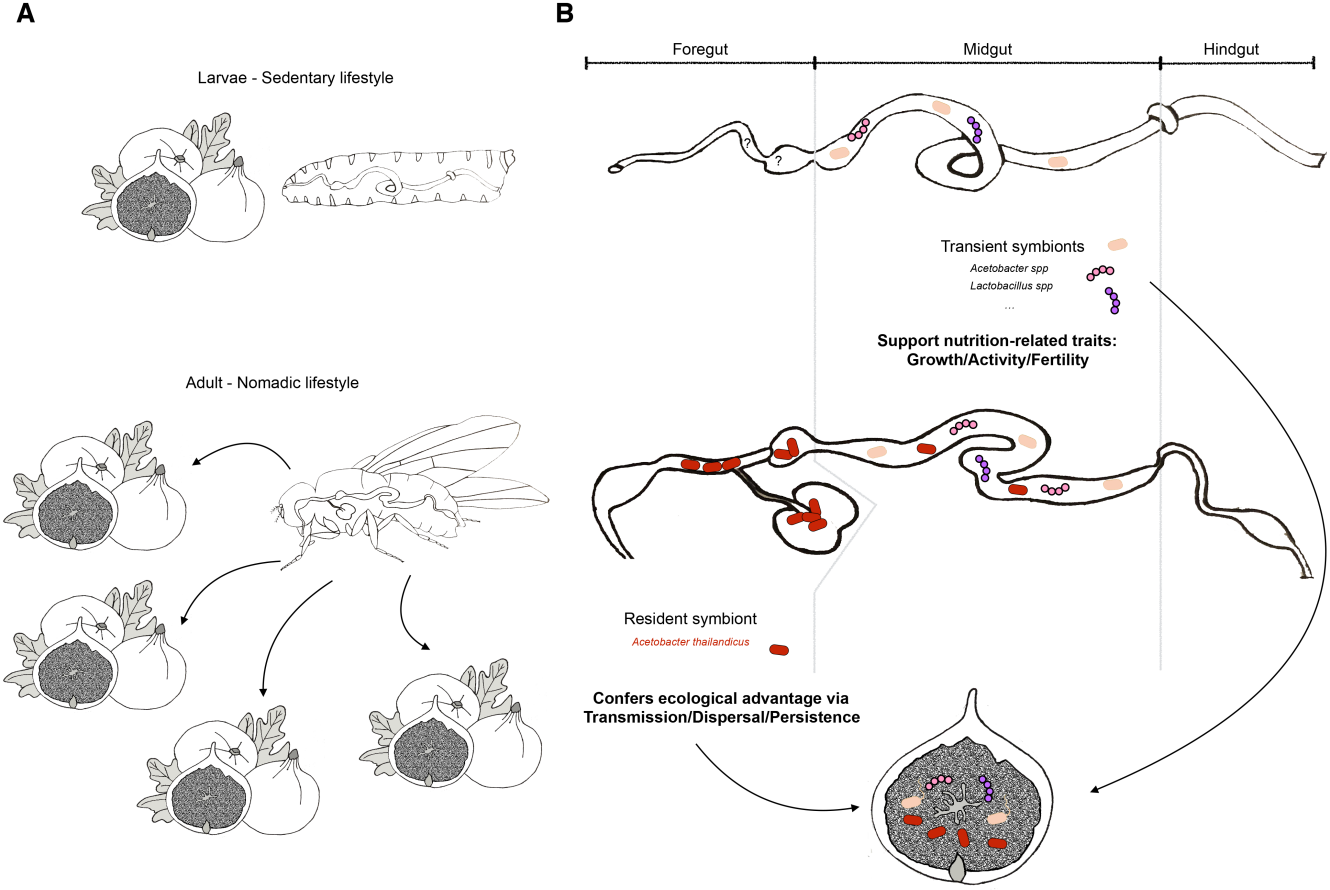
In this issue of *PLOS Biology*, Pais and colleagues isolate the first true resident gut symbiont of *Drosophila melanogaster*. This discovery renders the *Drosophila* model even more versatile and conducive to studying the different modalities of symbiosis. (A) Along its life cycle, *Drosophila* adopts two radically different lifestyles; larvae (and their symbionts) are sedentary in the ripe fruits where females deposit their eggs. In contrast, adults are nomadic, wandering from fruit to fruit, hence dispersing their progeny and their symbionts into multiple new niches. (B) Based on the inherently different foraging behavior and niche choice, the symbiotic modalities in larvae and adults differ. Larvae are associated with transient symbionts constantly acquired through ingestion of contaminated fruits and regularly shed back into the same substrate. These transient symbionts are found in the lumen of the midgut, where digestion and nutrients absorption take place. They effectively support the digestive and metabolic potential of the host and nutritionally enrich the food substrate thereby benefiting the host’s nutritionally related traits such as growth and maturation. In adults, the transient symbionts are also found in the midgut, which support nutrition-related traits such as fertility. In addition, Pais and colleagues identify a resident population of *Acetobacter thailandicus* in the foregut, the anterior part of the intestinal tract. Therefore, despite irregular and inconstant food intake and quality, the adults carry a persisting symbiont community in the gut that acts as a seed population to inoculate beneficial microbes onto new food substrate through excretion and thereby confers ecological advantages to the host via robust transmission to the progenies. *Credits for larvae*, *adult fly*, *and fruit drawings*: *Vincent Raquin*, *IGFL*. IGFL, Institut de Génomique Fonctionnelle de Lyon; spp, species.

What are the physiological consequences of having a stable gut symbiont for *Drosophila*? Pais and colleagues addressed how stable association with *A*. *thailandicus* affects two of the fly host’s fitness parameters: duration of development and fertility. Expectedly, on fresh figs, *A*. *thailandicus*–associated larvae develop slightly faster and are much more fertile. This likely reflects that *A*. *thailandicus* is more adapted to the natural food substrate where it was first isolated. On other food substrates, the presence of *A*. *thailandicus* accelerates maturation and increases viability to the same extent that the unstable *Acetobacter* OTU 2753 does. Interestingly, the physiological advantage conferred by *A*. *thailandicus* becomes readily apparent in the F_1_ generation, whose parents were associated at the embryonic stage, in that the progeny develop faster and lay more eggs compared to the *Acetobacter* OTU 2753–associated F_1_ progeny, who behave like the axenic flies. This is, however, not surprising. The frequent transfer protocol favors a stable colonizer in the foregut, which can act as a reliable depository that constantly replenishes the food and ensures the efficient transfer of the bacteria from parents to offspring; the stringency also disfavors any gut-unstable strain like *Acetobacter* OTU 2753 and prevents them from persisting and therefore becoming beneficial. The greater transgenerational fecundity and viability is likely attributed to sheer bacteria quantity rather than to profound physiological alterations in the host.

The first pressing question is how prevalent stable association is in nature. The relative abundance of *A*. *thailandicus* in the initial collective pool of the natural gut isolates is low (around 1%), yet 90% of the wild-captured flies carried it, and therefore, it is difficult to infer if stable association is an episodic occurrence or a predominant phenomenon in nature. Next, as the authors proposed, it will be interesting to assess how stable association has been lost in the lab-reared flies. The answers to these questions will uncover the fundamental requirements for the establishment and maintenance of the different mutualism modalities, the contribution of the genotype–environment interactions to the modalities, and the selection pressure and tradeoffs associated with adopting these modalities. As more and more gut resident strains are identified, metagenome-wide association studies, experimental evolution, and random mutagenesis can be deployed to pin down the genetic components for stable association from both the symbiont and the host. Interestingly, *A*. *thailandicus* does discriminate who the host is: it prefers *D*. *melanogaster* to *D*. *simulans*, and it colonizes the laboratory *w*^*1118*^ strain more efficiently than the naturally derived fly strains, indicating that host genetics does also play a role in colonization.

Next, whether natural strains can stably colonize the larval gut is also an interesting and multilayered question, even though the larva’s distinct ecological niche, physiological needs, and developmental program seem to strongly preclude this possibility. The larvae forage within a confined spot selected by their parents and feed constantly to meet their extraordinary growth demand (Fig 1A). As the number of bacteria is always greater in the food than in the gut, the bacteria that can transit through the gut more rapidly and proliferate better on the food will likely overtake the niche. In this ecological context, the farming mechanism maintains a constant and robust flux, and persistence is thus not a prerequisite for the mutualistic cycle (Fig 1B). On the other hand, the adult parents barely eat but move frequently to sample different food substrates for egg laying, so they are more likely to encounter a hostile environment that resembles the “stability protocol” setup employed by Pais and colleagues. Thus, harboring an inoculum of resident beneficial microbe for ready dispersal is a clear advantage for the next generation and for the bacteria. Therefore, different ecological niches, feeding behaviors, and physiological needs probably have shaped the distinct modalities of symbiosis adopted by the larvae and the adults (Fig 1). In addition, the larval gut is an entirely different habitat than the adult gut. The larval foregut lacks a structure orthologous to the crop, and the midgut is an extremely hostile environment that decimates the majority of the ingested bacteria [39]. Most strikingly, during metamorphosis, the larval gut is eliminated along with its microbes, thus rendering microbial persistence unlikely through this process. However, finding mutations that adversely affect the attachment of *A*. *thailandicus* to the adult crop and proventriculus will significantly advance our understanding in crop formation and function. In addition, should *A*. *thailandicus* persist in the transient larval or pupal gut through a shielded domain, studies that both elucidate and disrupt the timing and location of such event will bring incredible insights to the deep understanding of the symbiosis embedded within the developmental and morphogenetic processes of gut growth and remodeling.

The identification of a bona fide resident gut symbiont is a game changer for the *Drosophila* symbiosis model—not that it invalidates the current model with unstable microbiota; rather, it renders the fly an even more versatile and conducive model to study different modalities of nutritional mutualism. An important observation in this study is that in standard laboratory conditions, including growth on fig homogenate, stable association with *A*. *thailandicus* confers no greater fitness advantage to the host than the unstable laboratory *Acetobacter* strain. Thus, available laboratory strains work equally well as a persistent strain to modulate nutrition-dependent host traits such as growth, maturation, and fecundity (Fig 1B). In this context, the unstable and persistent associations are likely to be equally valuable. In fact, the discovery of *A*. *thailandicus* persistence now enables researchers to elucidate how stable association in comparison to or in combination with unstable strains can affect different fitness parameters that are not yet addressed in this study, such as stress resistance, immune response, longevity, and health span. Consequently, the unstable laboratory strains for studying microbial impact on host physiology are rendered even more multifaceted and pertinent.

Clearly, to study the mechanism of persistence and its evolutionary and ecological implications on symbiosis, *Drosophila* monoassociated with a resident gut commensal is a novel model with enormous potential. First, as the fly host harbors a stable population of beneficial gut microbes that can be vertically transferred to the next generation, this model can now be readily adapted to study host–microbiome coevolution in different environmental conditions. Secondly, stable association with the host promotes efficient dispersal and invasion of different ecological niches by the bacteria. As a result, the stable association model will be a valuable tool to dissect the complex microbial interactions among species such as ecological succession, resource competition, and cooperation within the context of symbiosis with a eukaryotic host. Therefore, to persist or not to persist, the choice is yours.

## Abbreviations

CFU: colony-forming unit
FISH: fluorescent in situ hybridization
IgA: immunoglobulin A
IGFL: Institut de Génomique Fonctionnelle de Lyon
OTU: Operation Taxonomic Unit
spp: species
TEM: transmission electron microscopy
